# Identification of tyrosine kinase inhibitors that halt *Plasmodium falciparum* parasitemia

**DOI:** 10.1371/journal.pone.0242372

**Published:** 2020-11-12

**Authors:** Kristina Kesely, Panae Noomuna, Michal Vieth, Philip Hipskind, Kasturi Haldar, Antonella Pantaleo, Francesco Turrini, Philip S. Low

**Affiliations:** 1 Department of Chemistry, Purdue University, West Lafayette, IN, United States of America; 2 Institute for Drug Discovery, Purdue University, West Lafayette, IN, United States of America; 3 Eli Lilly and Company, San Diego, CA, United States of America; 4 School of Medicine, Indiana University, Bloomington, IN, United States of America; 5 Clinical Pharmacology R2 402 MDEP, Indianapolis, IN, United States of America; 6 Galvin Life Science Center, University of Notre Dame, Notre Dame, IN, United States of America; 7 University of Sassari, Sassari, Italy; 8 University of Torino, Torino, Italy; Institut national de la santé et de la recherche médicale - Institut Cochin, FRANCE

## Abstract

Although current malaria therapies inhibit pathways encoded in the parasite’s genome, we have looked for anti-malaria drugs that can target an erythrocyte component because development of drug resistance might be suppressed if the parasite cannot mutate the drug’s target. In search for such erythrocyte targets, we noted that human erythrocytes express tyrosine kinases, whereas the *Plasmodium falciparum* genome encodes no obvious tyrosine kinases. We therefore screened a library of tyrosine kinase inhibitors from Eli Lilly and Co. in a search for inhibitors with possible antimalarial activity. We report that although most tyrosine kinase inhibitors exerted no effect on parasite survival, a subset of tyrosine kinase inhibitors displayed potent anti-malarial activity. Moreover, all inhibitors found to block tyrosine phosphorylation of band 3 specifically suppressed *P*. *falciparum* survival at the parasite egress stage of its intra-erythrocyte life cycle. Conversely, tyrosine kinase inhibitors that failed to block band 3 tyrosine phosphorylation but still terminated the parasitemia were observed to halt parasite proliferation at other stages of the parasite’s life cycle. Taken together these results suggest that certain erythrocyte tyrosine kinases may be important to *P*. *falciparum* maturation and that inhibitors that block these kinases may contribute to novel therapies for *P*. *falciparum* malaria.

## Introduction

Malaria remains a major cause of death in much of the underdeveloped world [1–6]. In 2018, there were 228 million new cases resulting in 405,000 deaths, mostly occurring in infants and the elderly [7]. Ninety percent of these cases occurred in sub-Saharan Africa where one child dies every two minutes from the parasitemia [7,8]. With multi-drug resistant strains emerging in Southeast Asia [9–15], and rates of infection beginning to increase again [8], interest in finding new cures for the disease is escalating [16–18]. Indeed, according to the World Health Organization, $2.7 billion was invested in malaria research in 2018 alone [7,19].

During previous studies of human erythrocyte membranes, we observed that tyrosine phosphorylation of the erythrocyte transmembrane protein, band 3, promotes dissociation of the spectrin-based membrane cytoskeleton from the lipid bilayer via a mechanism that involves an intramolecular association of the phosphorylated cytoplasmic domain of band 3 (cdb3) with an SH2-like (MESH) sequence within the membrane-spanning domain of band 3 [20,21]. Because this phosphorylation-induced cytoskeleton dissociation was found to cause membrane vesiculation and fragmentation [20–22], and since band 3 was observed to become increasingly tyrosine phosphorylated during maturation of *Plasmodium falciparum* within infected erythrocytes [23,24], we hypothesized that egress of the parasite from its red blood cell (RBC) host might require the parasite-stimulated tyrosine phosphorylation of band 3 in order to weaken the RBC membrane in preparation for parasite escape. A subsequent search for possible *P*. *falciparum* tyrosine kinases that might perform this membrane-weakening function, however, yielded no obvious tyrosine kinase gene candidate in the *P*. *falciparum* genome [25,26], suggesting that the band 3 tyrosine phosphorylation might be performed by a red blood cell encoded tyrosine kinase. When considered together with previous data showing that escape of *P*. *falciparum* from the infected RBC at the end of the parasite’s intra-erythrocyte life cycle could be prevented by inhibiting the tyrosine phosphorylation of band 3 [24], it seemed reasonable to posit that activation of an erythrocyte kinase might be required for normal parasite maturation.

In an effort to pursue a more unbiased approach towards identifying protein tyrosine kinase (PTK) inhibitors with anti-malaria activity, we screened a blinded library of kinase inhibitors from Eli Lilly and Co. for their abilities to prevent proliferation of *P*. *falciparum* in human blood cultures in vitro. We report here that Syk kinase inhibitors can prevent merozoite egress from late stage (schizont) *P*. *falciparum* infected RBCs, and that other classes of kinase inhibitors either have no effect on *P*. *falciparum* propagation or block parasite maturation at other stages of the parasite’s life cycle. Because none of the currently used anti-malaria drugs act on PTKs, it is conceivable the one of the PTK inhibitors identified here could constitute the starting point for development of an orthogonal therapy for malaria. Although several researchers have previously reported that kinase inhibitors can suppress parasitemia while others have emphasized the need to develop a host-targeted strategy for treatment of malaria [27–32], none of these studies have defined their mechanisms of action nor determined that inhibition of band 3 tyrosine phosphorylation and the consequent erythrocyte membrane destabilization can prevent parasite egress from the infected erythrocyte.

## Methods

### Processing of blood samples

All blood samples were obtained from healthy volunteers via venipuncture following informed consent using procedures approved by the Purdue University Institutional Review Board and conducted in accordance with Good Clinical Practice guidelines and the Declaration of Helsinki. Blood donors were Purdue University individuals (staff members and students) drawn from varying races and genders. The consent form stipulates clearly that the donation is entirely voluntary, and participants may decline to participate or withdraw from participation at any point without any penalty. No blood quality control procedures were performed on donor blood samples to test for blood pathogens.

### Preparation of compounds

Forty compounds received from Eli Lilly and Co. were provided in 10 mM DMSO solution in 96-well plates and stored at -20°C until use. Each compound was identified using the last 2 digits of the plate identification code (i.e. plate K000362926 was referred to as plate 26) plus the well position in which the compound was located on the 96-well plate (i.e. 26 A2, refers to the compound on plate 26 located in well A2). These compounds were selected based on their structural similarity to tyrosine kinase inhibitors provided to Eli Lilly, to find analogs of PTKs and novel antimalarial compounds. Dilutions of each compound were prepared in DMSO and added directly to culture plates prior to addition of parasite cultures. Each compound was assessed at concentrations of 1 μM and 10 μM in duplicate wells. The amount of DMSO used in the parasite cultures during kinase treatment was always kept under 0.5% v/v. Compounds with the ability to suppress parasitemia at 1 μM were considered potent, whereas compounds that only suppressed parasitemia at 10 μM were labeled as weak inhibitors. The compounds’ structures were initially blinded until all assays in the study were completed and all results analyzed to prevent any bias.

### Preparation and synchronization of *P*. *falciparum* cultures

After donation, blood was immediately processed as previously described [21]. Briefly, RBCs were separated from plasma and leukocytes by three washings in wash medium [RPMI 1640 (Invitrogen) containing 2 mM glutamine, 25 mM HEPES, 20 mM glucose, 27 μg/mL hypoxanthine and 32 μg/mL of gentamicin (Sigma) (pH 7.2)]; *P*. *falciparum* strain Palo Alto was then cultured at 1–5% hematocrit [33] under a 1% O_2_, 5% CO_2_, and 94% N_2_ atmosphere in complete media (CM) [wash medium supplemented with 0.5% Albumax II (Gibco)]. Parasites were synchronized using a Percoll gradient method, as previously described [24]. After allowing the synchronized parasites to mature and reinvade fresh RBCs, drug studies were performed at the desired times as hours post-invasion (hpi). To assess parasitemia and infected cell morphology, thin smears were prepared, labeled with Diff-Quick stain (Siemens), and examined by light microscopy.

### Susceptibility and phenotype evaluation of treated *P*. *falciparum* cultures

After allowing the synchronized parasites to mature and reinvade fresh RBCs, ring stage infected cultures at 2% hematocrit (hct) (0.5–1% parasitemia) in CM were treated with the compounds provided by Eli Lilly described above. Healthy erythrocytes used for culturing and experimentation were collected from the same donor to reduce any possible donor-related variations. Analysis time points included 24 h after treatment to evaluate growth inhibition and 48 h after treatment to evaluate reinvasion efficiency. At the specified time points, aliquots of the desired culture were removed and stained with SYBR Green I DNA stain prior to analysis by flow cytometry to determine level of parasitemia (DNA abundance) per cell. Data was analyzed using Flow Jo. To assess parasitemia and infected cell morphology, thin smears were prepared, labeled with Diff-Quick stain (Siemens) and examined by light microscopy.

### Induction of tyrosine phosphorylation of uninfected RBCs with o-vanadate and diamide

Blood collected from healthy volunteers as described above was centrifuged to remove the plasma and buffy coat layer (containing white blood cells). Red cells were then washed thrice with phosphate buffered saline [137 mM NaCl, 2.7 mM KCl, 8 mM Na_2_HPO_4_, and 2 mM KH_2_PO_4_, pH 7.4] supplemented with 5 mM glucose (PBS-G) and resuspended in PBS-G at 30% hct prior to treatment with the desired kinase inhibitors in a final DMSO concentration of 0.5% v/v. Samples treated solely with either orthovanadate (OV) or diamide served as positive controls while samples treated solely with DMSO served as negative controls. After addition of inhibitors, samples were incubated for 1 h at 37°C with shaking, after which 2 mM orthovanadate or 2 mM diamide was added to induce band 3 tyrosine phosphorylation. The samples were again incubated for 1 h at 37°C with shaking and then centrifuged to collect the erythrocytes. The pelleted erythrocytes were then lysed by resuspending in 10 volumes of ice-cold lysis buffer [5 mM Na_2_HPO_4_, 1 mM EDTA, 0.5 g/100 mL sodium azide (pH 8.0)] supplemented with 1 mM phenylmethylsulfonyl fluoride (PMSF), phosphatase inhibitors cocktails 2 and 3, and protease inhibitor cocktail (Sigma) (added immediately before use) for 10 minutes and the resulting membranes were collected by centrifugation at 0°C. After aspiration of the supernatant, the remaining RBC ghosts were washed another 3 times with cold lysis buffer/1mM PMSF and centrifuged. The pelleted ghosts were solubilized in 2X Laemmli sample buffer (2% SDS) containing 5% 2-mercaptoethanol and 1mM PMSF and incubated for 30 min prior to storage at -80°C until analysis.

### Quantitation of the inhibition of malaria-induced band 3 tyrosine phosphorylation by Western blotting

To analyze inhibition of band 3 tyrosine phosphorylation by certain Eli Lilly kinase inhibitors, synchronized ring stage cultures at 20% parasitemia were removed, washed with CM and resuspended at 1% hct in CM prior to addition of 5 μM (final concentration) inhibitor. After incubation for 18 hours under a 1% O_2_, 5% CO_2_, and 94% N_2_ atmosphere in CM [24,33], the treated cells were transferred into 1.5 mL tubes and pelleted. Supernatants were removed and ice-cold lysis buffer was added to the 1.5 mL mark of each tube prior to incubation on ice for 30 minutes. Ghost membranes were prepared as described above, solubilized in 4X Laemmli sample buffer (2% SDS) containing 0.5 mM dithiothreitol (DTT) and 1mM PMSF, and incubated for 30 minutes at 45°C prior to storage at -20°C until SDS-PAGE analysis.

For SDS-PAGE analysis, samples were first thawed and then incubated at 95°C for 5 min. Samples were loaded onto a 10% polyacrylamide gel, subjected to electrophoresis, and transferred onto nitrocellulose membranes for Western blotting. Nonspecific binding was blocked by incubating membranes overnight in 5% non-fat milk–TBST [25 mM Tris, 140 mM NaCl, 3 mM KCl, 0.5% (v/v) Tween-20, pH 8.0] at 4°C with rocking. Membranes were then probed with anti-p-Tyr (PY99) (1:1000; mouse monoclonal; Santa Cruz Biotechnology # SC-7020) and anti-actin (1:10,000; rabbit polyclonal; Sigma Aldrich #A2103) antibodies in TBST for 1h at room temperature (RT) with gentle agitation. After washing, the membranes were incubated in secondary antibodies (1:10,000 anti-rabbit IgG, HRP-linked or 1:10,000 anti-mouse IgG, HRP-linked antibody according to the isotype of the primary antibody; Jackson ImmunoResearch Laboratories Inc. #715-035-150(Mouse) or #711-035-152(Rabbit)) for 30–60 min at RT with shaking and subsequently washed with TBST. When needed, a specific anti-phosphotyrosine antibody capable of detecting phosphorylated tyrosine 8 of band 3, was used in place of the nonspecific anti-phosphotyrosine antibody at 1:5000 dilution. The anti-phosphotyrosine 8 antibody was prepared in our lab with the help of Proteintech Inc. (Proteintech; antigen name: Li2760-EC1). Mouse monoclonal anti-band 3 antibody was obtained from Sigma Chemical Co. (#B9277) and used for band 3 staining at 1:10,000 dilution. Proteins were visualized by incubation with chemiluminescent substrate on a ChemiDoc Imaging System using Image Lab software (Bio-Rad).

## Results

**Fig 1:**
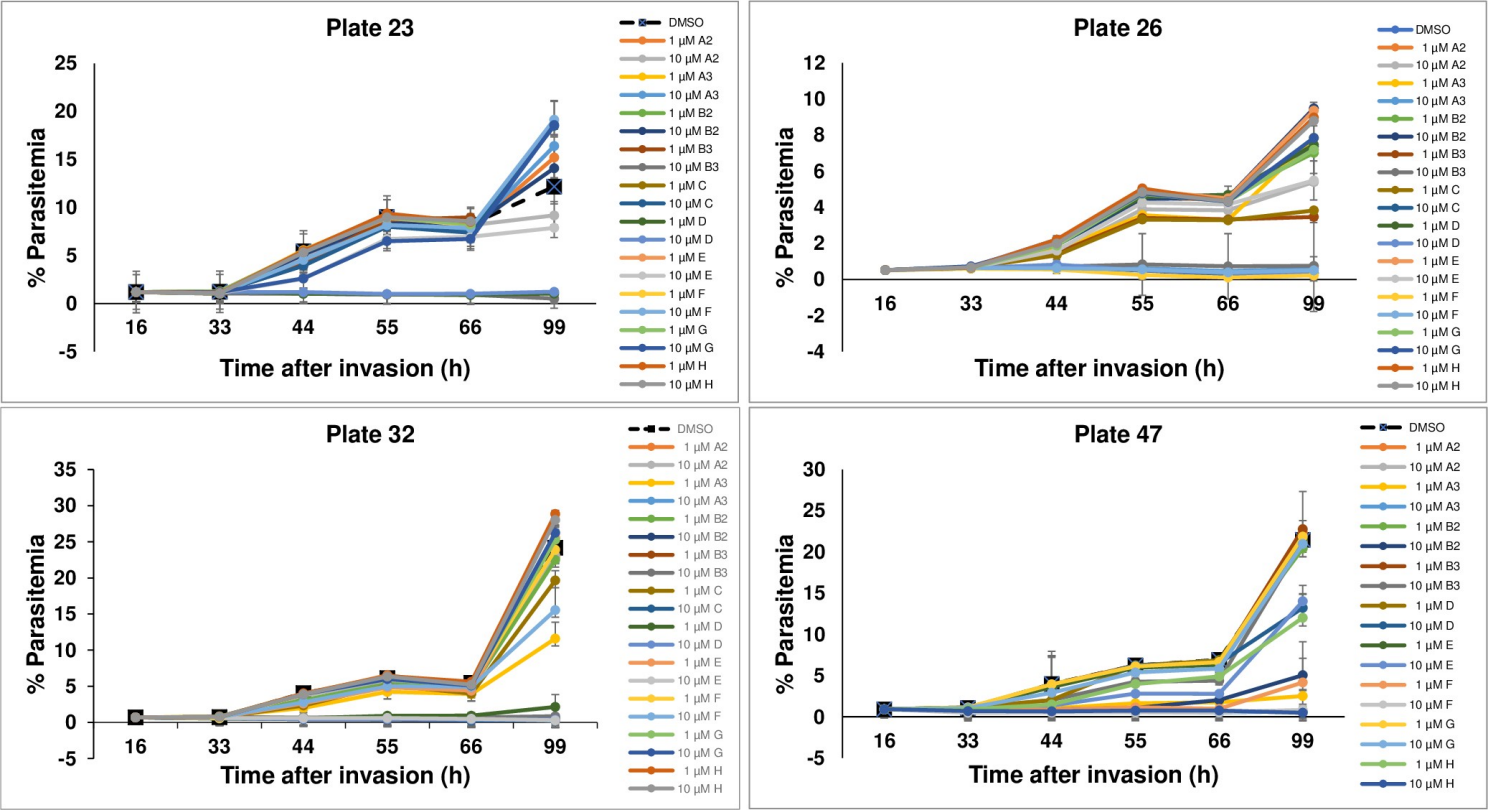
Percent parasitemia of *P*. *falciparum* infected RBCs as a function of time following treatment with de-identified tyrosine kinase inhibitors at 1μM and 10μM concentrations. The drugs were encoded based on their locations in 96-well plates as explained in the Methods section. Cultures were synchronized as described in Methods and kinase inhibitors were added ~16 hours after RBC invasion. The drug treatments were conducted in duplicate.

**Fig 2:**
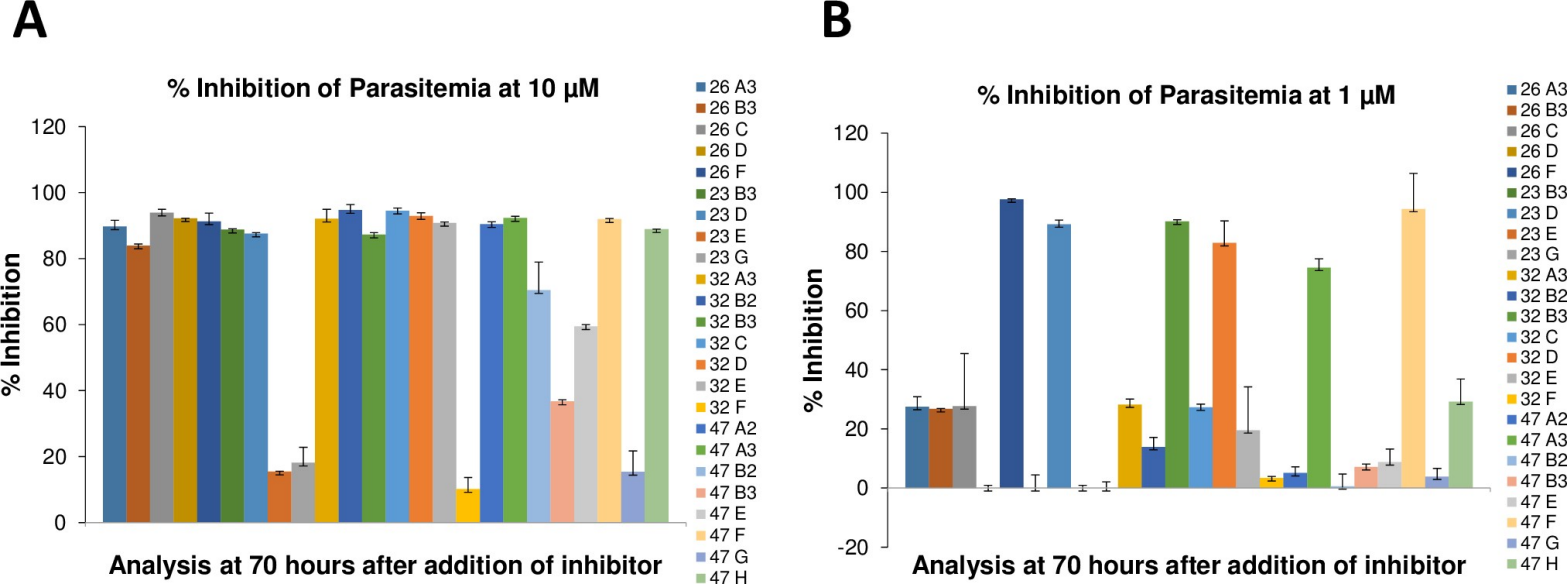
Percent inhibition of malaria parasitemia in RBCs following treatment with the most potent Eli Lilly compounds administered at 10 μM (panel A) and 1 μM (panel B) concentrations. The indicated per cent inhibition was determined at the 22 hours post invasion time point in the parasite’s second life cycle (at the 70 hour time point in Fig 1), when control cultures were at the mature ring stage of development. All the treatments were done in duplicate.

**Fig 3:**
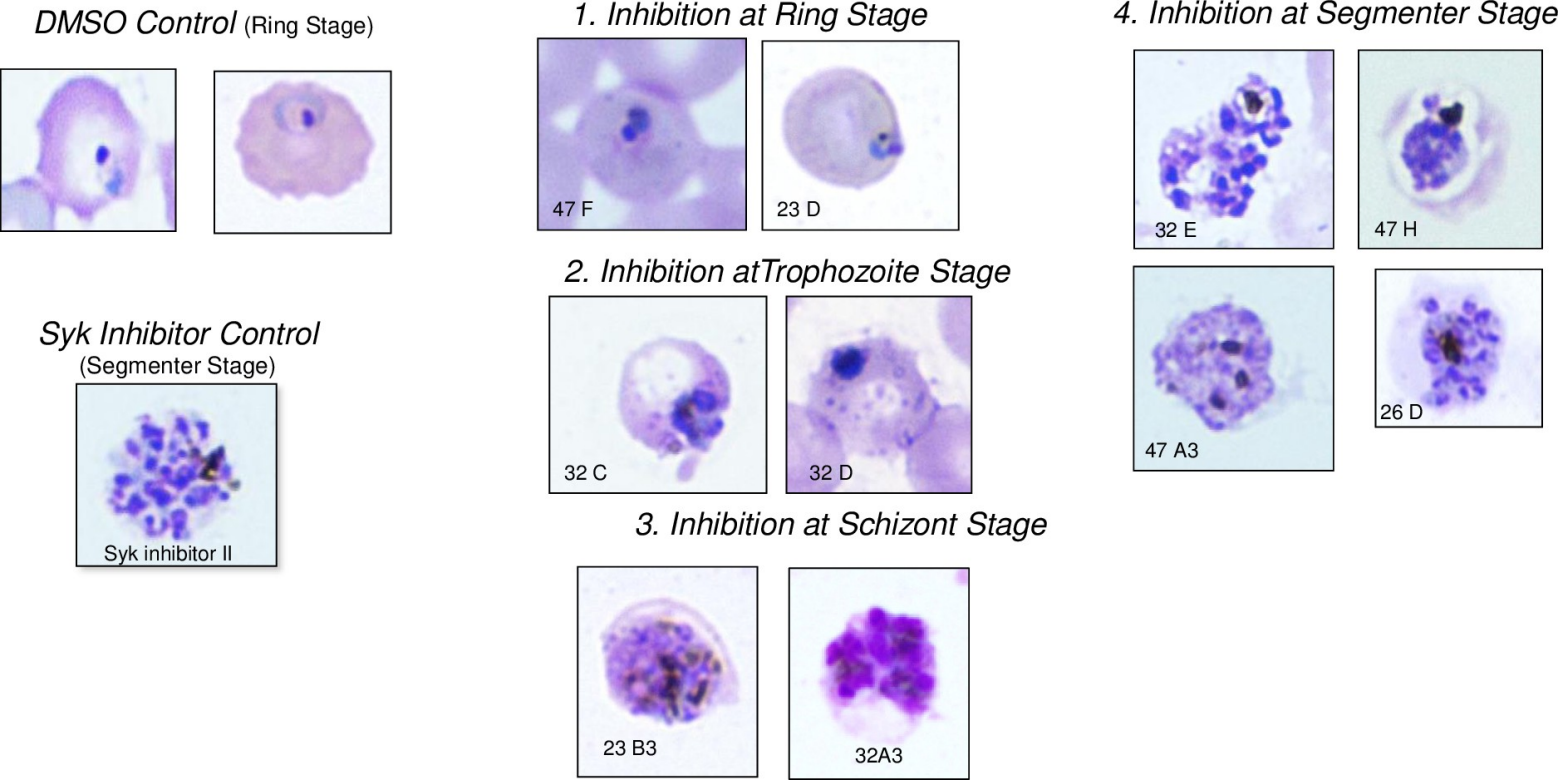
Images of blood smears of parasite cultures obtained 66 hours post invasion. Top left: Untreated (DMSO control) showing infected RBCs have progressed to the ring stage of their second life cycle after successful egress at the end of their first life cycle). Bottom left: Positive control of parasites treated with the well-known Syk inhibitor II showing stalled egress at the end of the first life cycle. Panels 1, 2, 3 and 4: Blood smears displaying the stages at which the de-identified drug terminated parasite maturation. Inhibitors 47F and 23D halted parasite maturation at ring stage, while 32C and 32D appear to halt the parasite at the trophozoite stage. Inhibitors 23B3 and 32A3 interrupted parasite development at the schizont stage. Syk phenotype inhibitors; 32E, 47H, 47A3 and 26D appear to stop the parasite from egressing at the segmenter stage as evidenced by the separated merozoites within infected cells.

In an effort to obtain an unbiased analysis of the effects of different PTK inhibitors on *P*. *falciparum* survival in human blood cultures, we established an Eli Lilly & Co.-Purdue University collaboration in which Eli Lilly researchers shared samples of 40 potent compounds from their tyrosine kinase inhibitor library [34–36] with Purdue researchers who then evaluated these compounds for inhibition of parasite propagation and band 3 tyrosine phosphorylation in a blinded manner. Each of the 40 kinase inhibitors was added to *P*. *falciparum* cultures at their ring stage of development and propagation of the cultures was monitored for 99 hours. As seen in Fig 1, most inhibitors displayed little to no effect on parasite propagation, prompting us to dismiss them from further consideration. In contrast, a few inhibitors on each plate were observed to either reduce or completely prevent parasite proliferation, with a subset of these inhibitors impacting parasite survival at only 10 μM concentration (Fig 2, panel A) and a more potent subset exhibiting efficacy at 1 μM (panel B). To obtain information on the stage of parasite development at which each inhibitor interrupted the parasite’s life cycle, inhibitor-exposed cultures that displayed suppression of parasite proliferation (Fig 2) were further examined microscopically for information on the stage of *P*. *falciparum* maturation where development was halted. In all cases, synchronized cultures were treated during their ring stage of development and examined over the following 48 hours. As shown in Fig 3, four different classes of inhibitors were identified based on these criteria. Although inhibitor-free cultures (DMSO controls) progressed through a normal life cycle in the typical 48 h time frame, cultures incubated with compounds 47F and 23D were found to die during the ring stage of their initial life cycle. In contrast, cultures exposed to compounds 32C and 32D were halted at the trophozoite stage of maturation and parasitized cells treated with 23B3 and 32A3 were interrupted at the schizont stage of development. Finally, cultures exposed to 32E, 47H, 47A3, and 26D all died during the segmenter stage of their first life cycle, i.e. the same stage observed in cultures treated with known Syk kinase inhibitor controls. These data imply that the different kinase inhibitors likely act on a spectrum of molecular targets that become critical to parasite development at varying stages of development.

**Fig 4:**
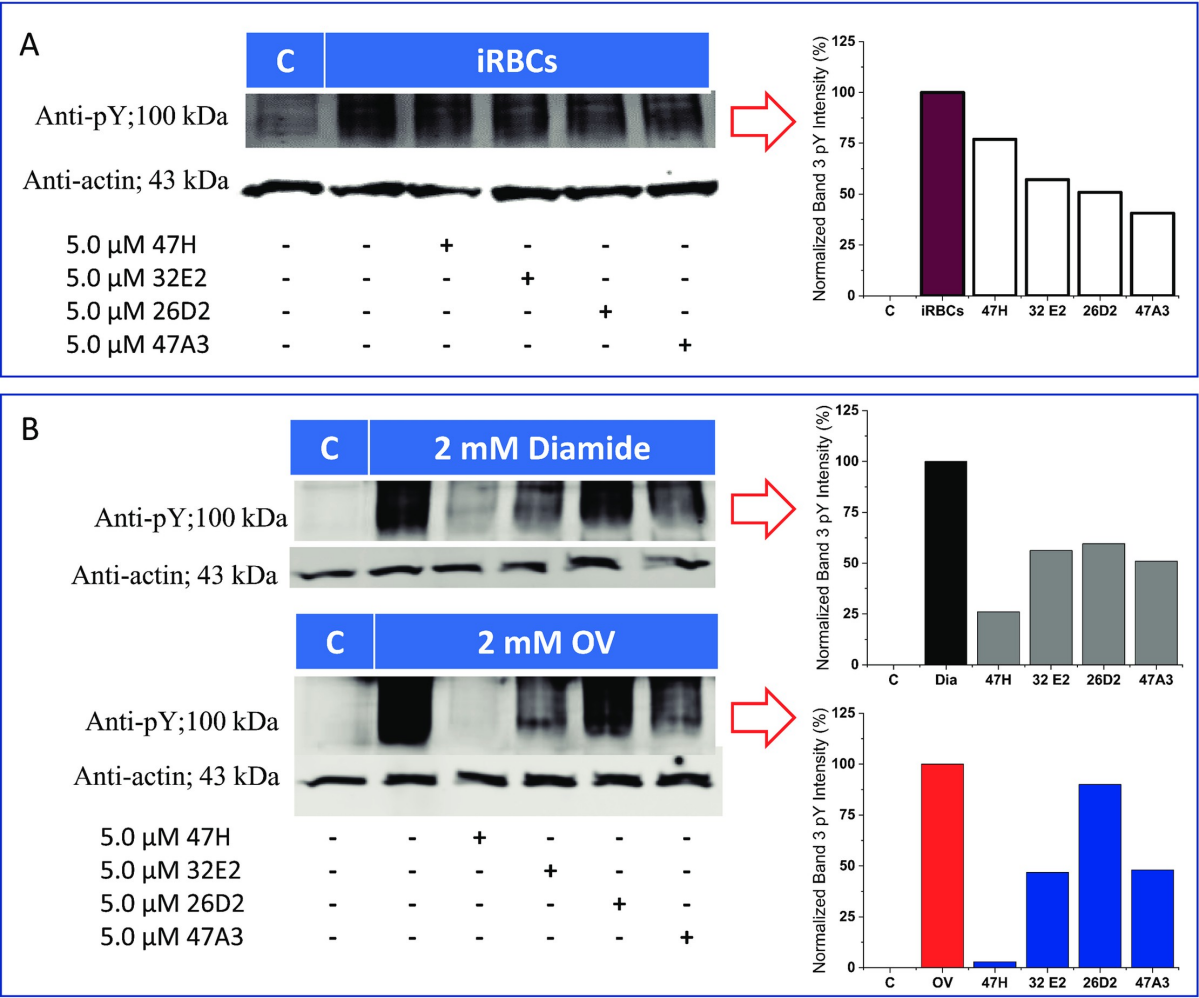
Western blots displaying band 3 tyrosine phosphorylation and its inhibition by the Eli Lilly compounds found to display the Syk inhibitor phenotype. All the selected inhibitors reduced parasite-induced band 3 tyrosine phosphorylation compared to controls (panel A). The compounds also inhibited diamide (top) and orthovanadate (bottom)-induced band 3 tyrosine phosphorylation, albeit with different potencies (panel B). It is worth noting that the inhibitors blocked diamide and orthovanadate-induced band 3 tyrosine with identical trends.

Because our initial hypothesis proposed that tyrosine kinase inhibitors that blocked phosphorylation of band 3 should prevent parasite egress at the schizont/segmenter stage of the parasite’s life cycle [24,37], we elected to explore whether the compounds that blocked parasite development at the egress stage (e.g. 47A3, 32E, 47H, and 26D) might also inhibit band 3 tyrosine phosphorylation. However, because intact band 3 and most other erythrocyte membrane proteins could not be resolved in SDS-PAGE gels at this late stage of parasite development [23,37], we were forced to evaluate inhibition of band 3 tyrosine phosphorylation at earlier stages of parasite maturation; i.e. where intact erythrocyte membrane proteins are more abundant and readily identified by SDS-PAGE. As shown in Fig 4A, all four schizont/segmenter stage inhibitors were found to reduce band 3 tyrosine phosphorylation at this earlier stage of parasite development. While two of the other inhibitors (47F and 23D; supplemental S2 Fig) were found to inhibit o-vanadate and diamide induced band 3 tyrosine phosphorylation, this inhibitory activity could not contribute to their anti-malaria potency, because they were found to interrupt the parasite’s life cycle at an earlier stage of development. To provide direct evidence that the strong phosphotyrosine signal detected at ~100 kDa indeed derives from band 3, we employed both an antibody specific for phosphotyrosine 8 on band 3 and an antibody that recognizes the protein band 3 to stain analogous immunoblots of malaria-infected RBCs. As shown in S3 Fig, the antibody specific for phosphotyrosine 8 on band 3 stains both orthovanadate-treated and malaria infected RBC membranes (panel A). Moreover, as demonstrated in panel B, this band co-migrates with the band that stains positive with the anti-band 3 specific antibody. We therefore conclude that the major tyrosine phosphorylated protein in the membranes of malaria-infected RBCs is erythrocyte band 3 and that erythrocyte band 3 is phosphorylated at least on tyrosine 8 of band 3 in malaria infected cells.

Next, to further confirm that these same four tyrosine kinase inhibitors are capable of suppressing the tyrosine kinase(s) that phosphorylate band 3 in situ, we treated healthy RBCs with two well-established inhibitors of erythrocyte tyrosine phosphatases (i.e. ortho-vanadate or diamide) that promote natural band 3 tyrosine phosphorylation by suppressing its constitutive dephosphorylation [38–44]. We then evaluated the abilities of these segmenter stage-specific tyrosine kinase inhibitors to block the endogenous phosphorylation of band 3. As shown in Fig 4B, all four inhibitors reduced both o-vanadate- and diamide-induced tyrosine phosphorylation of band 3 in a similar manner, albeit with different potencies. More importantly, the order of potencies for inhibition of band 3 tyrosine phosphorylation (47H≥47A3>32E2>>26D2) correlated crudely with the ranking of potencies for inhibition of parasite egress (47A3>32E2≥47H> >26D2) (see also IC_50_ values in supplemental S1 Fig panels A and B), arguing that the four identified PTK inhibitors likely function at least in part by suppressing tyrosine phosphorylation of band 3, as proposed previously [37].

That inhibition of other kinases might also contribute to the anti-malarial activities of these suspected tyrosine kinase inhibitors is suggested by the fact that potent inhibitors of other stages of *P*. *falciparum* development had no effect on band 3 tyrosine phosphorylation (e.g. 23B3, 26A3, 26B3, 32A3, 32B2, 32D, 32C and 47F; Figs 1 and 2, and supplemental information S2 Fig), suggesting that inhibition of other tyrosine kinases can also terminate the parasitemia. Because no known tyrosine kinase inhibitors have been found to be specific for a single tyrosine kinase, the identities of these other kinases could not be ascertained from the specificities of the inhibitors.

Upon completion of our blinded analysis of the effects of these PTK inhibitors analogs on parasite maturation, we submitted our ranked list of the 10 most potent anti-malaria kinase inhibitors to our Eli Lilly collaborators for annotation with chemical identities and primary kinase target identification [36,45–50]. Data from the annotation revealed that all potent inhibitors of *P*. *falciparum* egress at the segmenter stage as well as inhibitors of diamide and o-vanadate induced band 3 tyrosine phosphorylation (Fig 4B) display Syk tyrosine kinase inhibitory activity. Moreover, all kinase inhibitors that terminated the parasite’s life cycle at earlier stages of parasite development (i.e. schizont, trophozoite or ring stage) were found to be promiscuous kinase inhibitors, displaying inhibitory activities against multiple families of both tyrosine and serine/threonine kinases (Table 1). Assuming that these molecules act by inhibiting kinases involved in parasite development, it would appear that multiple kinases must perform important functions during *P*. *falciparum* proliferation and maturation. It can also be concluded that erythrocyte Syk-mediated weakening of the RBC membrane likely constitutes a critical step in the egress of mature merozoites from their erythrocyte hosts.

**Table 1:** Structures of tyrosine kinase inhibitors used in this study.

Drug Code	Structure	Class of Inhibitor	Most active kinase
**47F**	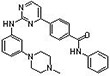	**Imatinib-Analog**	**Aurora A kinase**
**23D**	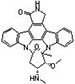	**Staurosporine**	**Broad spectrum kinase inhibitor**
**32C**	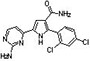	**No syk activity**	**CDC7, promiscuous**
**32D**	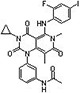	**No syk activity**	**MAP2K1, promiscuous**
**32A3**	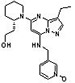	**No syk activity**	**CDK9, promiscuous**
**32E**	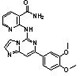	**Syk inhibitor IV**	**Syk**
**47H**	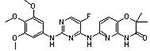	**Syk inhibitor R406**	**Syk**
**47A3**	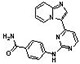	**Syk inhibitor IV analog**	**Syk, PI-3 kinase**
**26D**	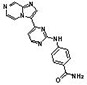	**Syk inhibitor IV analog**	**Syk, PI-3 kinase**
**23B3**	**Proprietary structure**	**No syk activity**	**CDC7, promiscuous**

## Discussion

Based on the above data and other publications from our labs [24,37], we conclude that erythrocyte Syk performs a critical function in the *P*. *falciparum* life cycle, and that its inhibition terminates the life cycle by preventing merozoite egress from the infected RBC. Data supporting this contention include the observations that i) band 3 tyrosine phosphorylation dramatically increases as parasite maturation progresses [23,24,37], ii) Syk is the major erythrocyte tyrosine kinase found to phosphorylate band 3 in vivo [21,51], iii) Syk is progressively activated and recruited to the RBC membrane as the parasite matures [23,52,53], iv) all known Syk inhibitors (and band 3 tyrosine phosphorylation inhibitors) display antimalarial activity [24,37], and v) incubation of parasite cultures with Syk inhibitors does not significantly alter earlier stages of parasite maturation even though the rise in band 3 tyrosine phosphorylation begins early and increases as the parasite progresses through its life cycle [24,37]. Collectively, these results argue that Syk kinase inhibitors interrupt a critical step required for parasite escape from its RBC host. The data also suggest that a potent and selective Syk inhibitor could constitute an excellent candidate for treatment of malaria.

Whether any of the inhibitors examined in this study directly kill the parasite or simply prevent its maturation is difficult to determine from the data. Thus, the supply of nutrients to the maturing parasite may be a limiting factor, especially as the hemoglobin and other RBC proteins are progressively consumed. Consequently, any drug that delays or halts parasite maturation could cause the parasite to die due to starvation. Moreover, once progression of the parasite through its life cycle is halted, toxic byproducts of parasite metabolism (e.g. unpolymerized heme) [54–56] could accumulate, rendering the intra-erythrocyte milieu increasingly incompatible with parasite survival, i.e. perhaps poisoning the parasite to death. With still other causes of *P*. *falciparum* death conceivable, the specific cause of death induced by tyrosine kinase inhibitors cannot be unambiguously defined. However, in can now be firmly concluded that inhibition of Syk can induce it.

Based on many previous studies, we wish to propose a sequence of events culminating in Syk inhibitor termination of *P*. *falciparum* parasitemia. Like diamide, consumption of hemoglobin and the concomitant release of heme creates a strongly oxidizing environment within the infected RBC [52,57–59]. Oxidation of active site cysteines in both major RBC tyrosine phosphatases then inhibits their activity [22,57,60–62], leading to stable band 3 tyrosine phosphorylation by constitutively active Syk. This tyrosine phosphorylation then promotes an intramolecular interaction between the phosphotyrosines on band 3 and an SH2-like domain within the membrane-spanning domain of band 3 [20] that in turn triggers dissociation of ankyrin and the spectrin-based RBC cytoskeleton from band 3 [21,63]. The resulting disjunction of the cytoskeleton from the membrane then causes the predicted weakening of the RBC membrane that allows parasite egress from the weakened RBC [24,37]. Inhibition of Syk catalyzed band 3 phosphorylation specifically blocks this weakening, preventing the escape of *P*. *falciparum* from its RBC host at the end of its life cycle. Based on these observations and the fact that the parasite cannot mutate an erythrocyte tyrosine kinase, we anticipate that a Syk kinase inhibitor, perhaps in combination with an artemisinin-like anti-malarial, might constitute a mutation-resistant therapy for malaria. Clinical trials currently underway in Vietnam and Laos should provide an accurate test of this hypothesis (see ClinicalTrials.gov; Identifier: NCT02614404 and/or NCT03697668).

## Supporting information

S1 Fig **A. Effect of inhibitor concentration on the percent of fresh erythrocytes that become infected following their co-incubation for 3 days with ring stage *P*. *falciparum* infected RBCs (Palo Alto strain). B. IC**_**50**_ **values of the selected inhibitors plotted in panel A.** The inhibitors for this study were chosen from the library of inhibitors examined in Fig 1 based on their potencies in suppressing parasitemia.
(TIF)

S2 Fig Evaluation of the ability of selected kinase inhibitors to suppress erythrocyte band 3 tyrosine phosphorylation induced by either diamide or o-vanadate. To determine whether all compounds in the Eli Lilly kinase inhibitor library that were found to have anti-malaria activity might also inhibit band 3 tyrosine phosphorylation, a selection of inhibitors with anti-malaria activity were examined for their abilities to suppress diamide or o-vanadate stimulated tyrosine phosphorylation of band 3. The anti-phosphotyrosine immunoblots of band 3 in membranes isolated from erythrocytes treated for 1 hour with drug followed by an additional hour treatment with either diamide (top panel) or o-vanadate (bottom panel) are shown. Inhibitor 23D blocked parasite development at the ring stage, 32D halted the life cycle at the trophozoite stage, and 23B3 interrupted maturation at the schizont stage, while 47H and 32E2 (both Syk inhibitors) were found to block development at the egress stage of the parasite’s life cycle.
(TIF)

S3 Fig Evidence that the intensely tyrosine phosphorylated band at 100kDa derives from band 3. Membranes from *P*. *falciparum*-infected (iRBCs) or orthovanadate (OV)-treated RBCs were analyzed by immunostaining with either an antibody specific for phosphotyrosine 8 on band 3 (anti-pY8) or a monoclonal antibody to whole band 3 (anti-band 3).
(TIF)

S1 Raw images Unadjusted immunoblots of the trimmed data presented in Fig 4A and 4B, S2 and S3 Figs. The lower half of each blot was stained with an anti-actin antibody (to establish that all lanes are loaded equally), while the upper half of each blot was stained with an antibody to the erythrocyte protein, band 3, or phosphotyrosine 8 on band 3, or any (nonspecific) phosphotyrosine.
(PDF)
