# Supplementary figures and images for: Identification of tyrosine kinase inhibitors that halt Plasmodium falciparum parasitemia
Source: PLoS One. 2020 Nov 12;15(11):e0242372. doi: 10.1371/journal.pone.0242372 (PMC7660480; doi:10.1371/journal.pone.0242372)

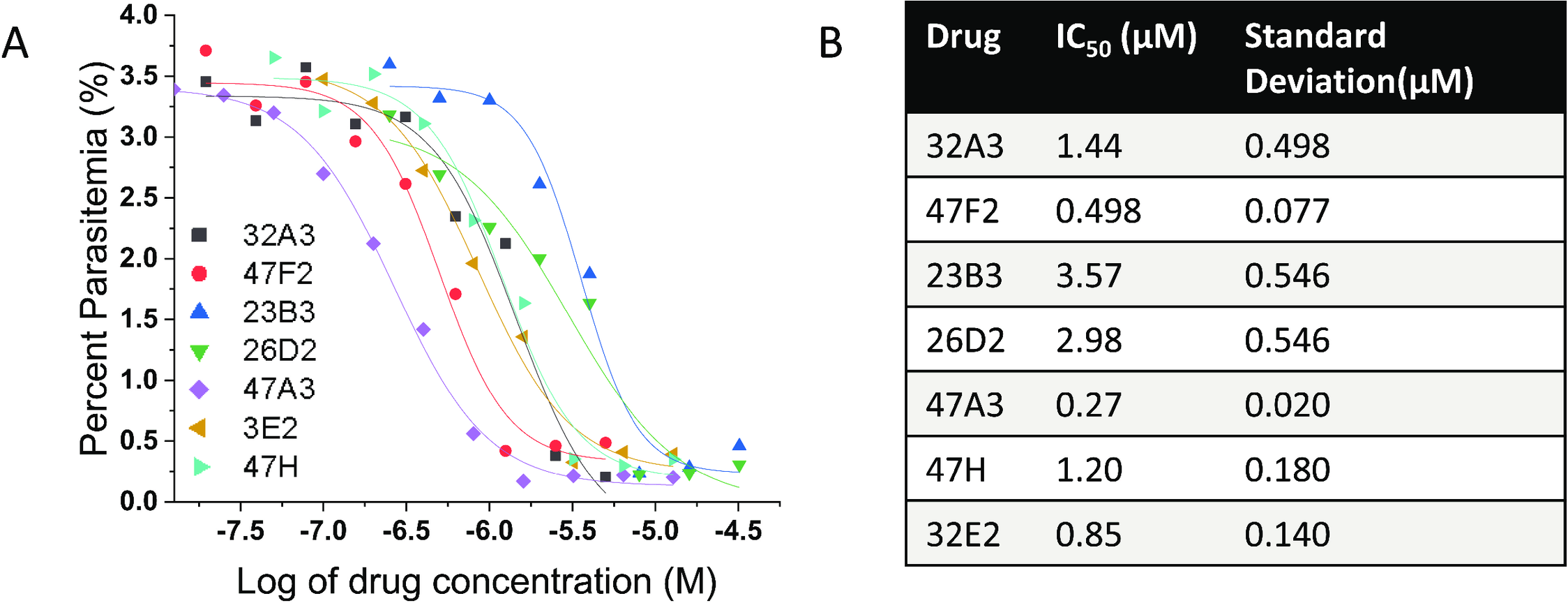

Supplement: S1 Fig — A. Effect of inhibitor concentration on the percent of fresh erythrocytes that become infected following their co-incubation for 3 days with ring stage P. falciparum infected RBCs (Palo Alto strain). B. IC50 values of the selected inhibitors plotted in panel A. The inhibitors for this study were chosen from the library of inhibitors examined in Fig 1 based on their potencies in suppressing parasitemia. (TIF) [file pone.0242372.s001.tif]

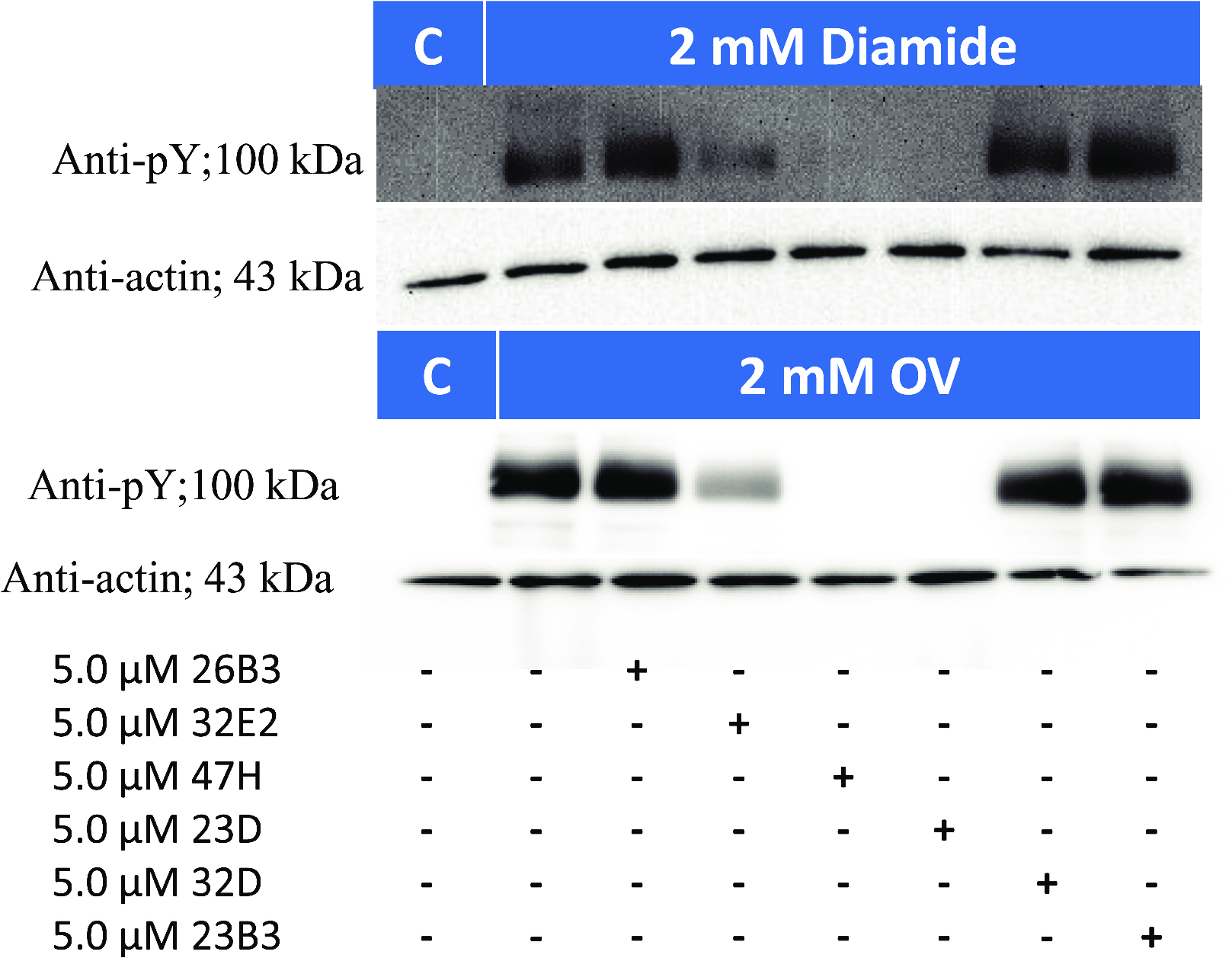

Supplement: S2 Fig — To determine whether all compounds in the Eli Lilly kinase inhibitor library that were found to have anti-malaria activity might also inhibit band 3 tyrosine phosphorylation, a selection of inhibitors with anti-malaria activity were examined for their abilities to suppress diamide or o-vanadate stimulated tyrosine phosphorylation of band 3. The anti-phosphotyrosine immunoblots of band 3 in membranes isolated from erythrocytes treated for 1 hour with drug followed by an additional hour treatment with either diamide (top panel) or o-vanadate (bottom panel) are shown. Inhibitor 23D blocked parasite development at the ring stage, 32D halted the life cycle at the trophozoite stage, and 23B3 interrupted maturation at the schizont stage, while 47H and 32E2 (both Syk inhibitors) were found to block development at the egress stage of the parasite’s life cycle. (TIF) [file pone.0242372.s002.tif]

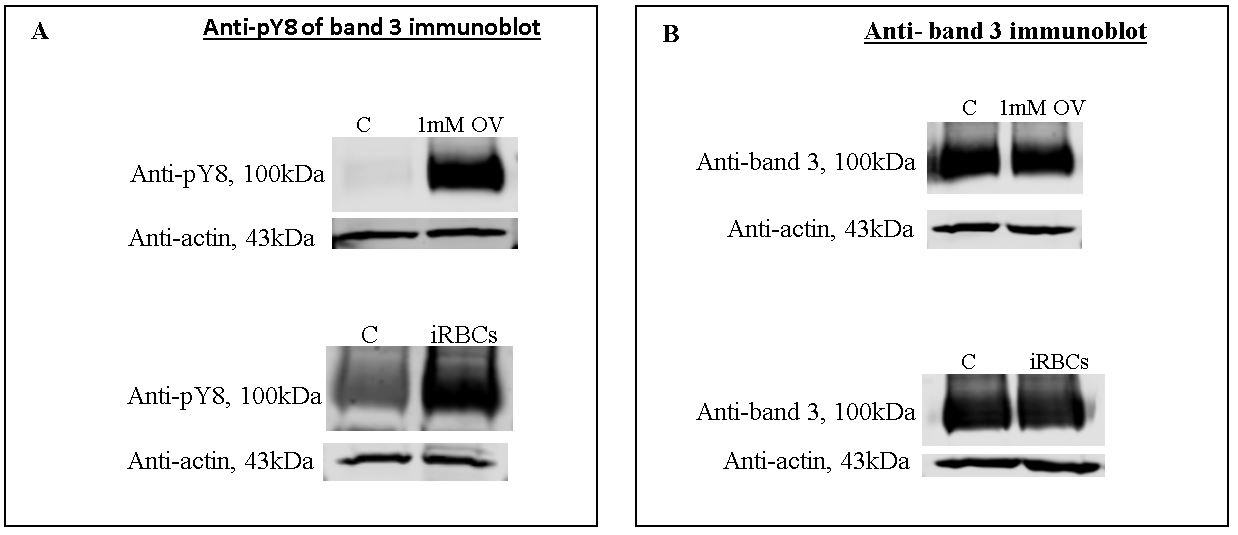

Supplement: S3 Fig — Membranes from P. falciparum-infected (iRBCs) or orthovanadate (OV)-treated RBCs were analyzed by immunostaining with either an antibody specific for phosphotyrosine 8 on band 3 (anti-pY8) or a monoclonal antibody to whole band 3 (anti-band 3). (TIF) [file pone.0242372.s003.tif]

Figure 4A: Unadjusted blots

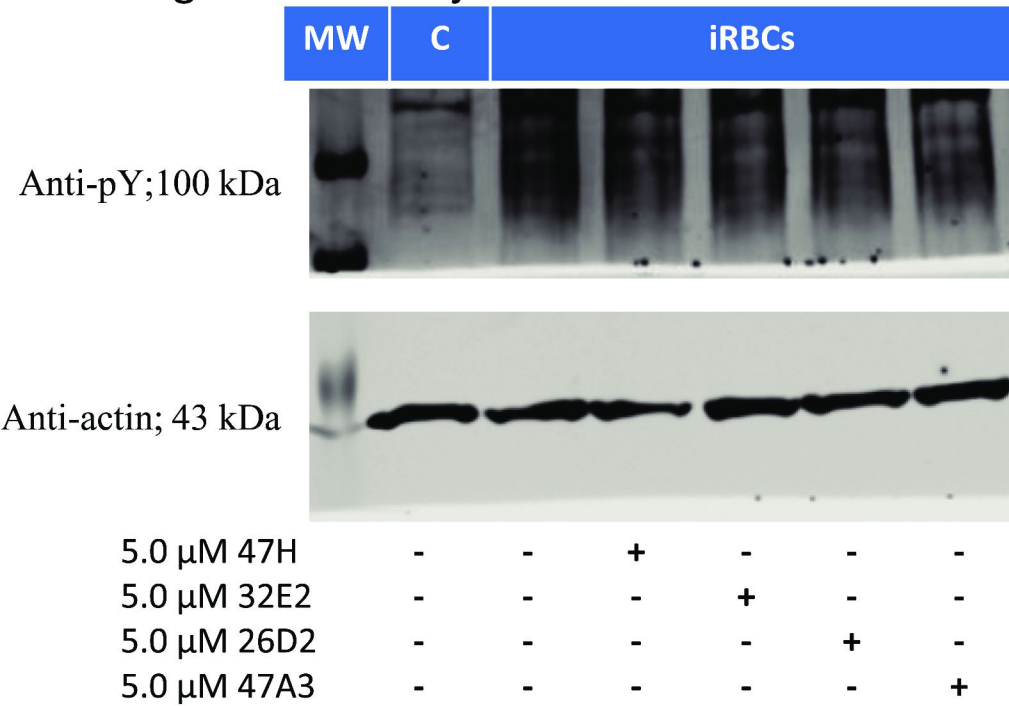

**Figure 4B: Unadjusted blots**

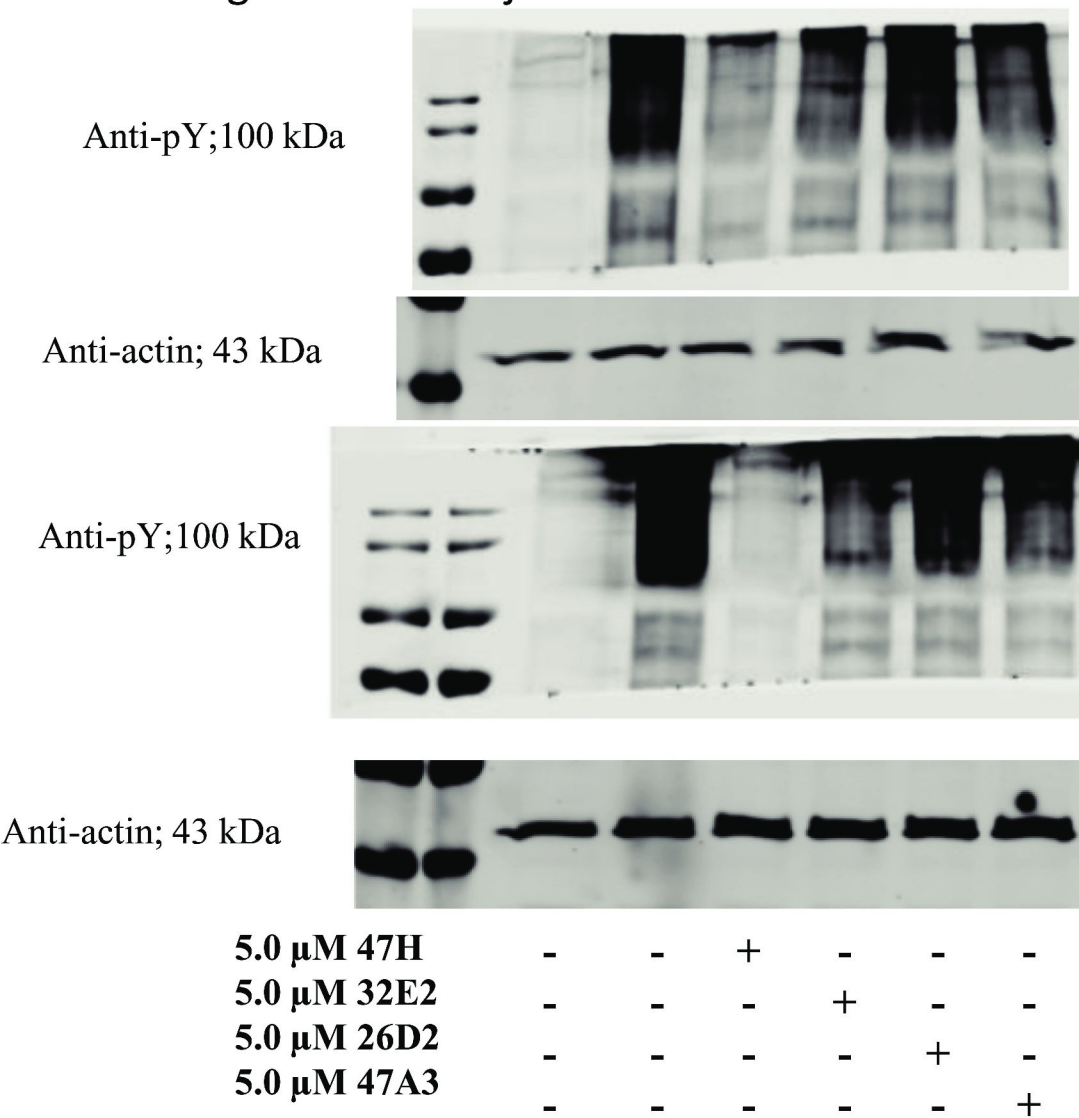



# S3 Fig: Unadjusted blots

**A**

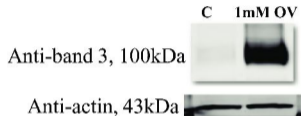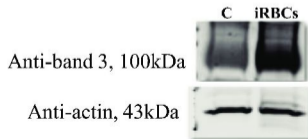

**B**

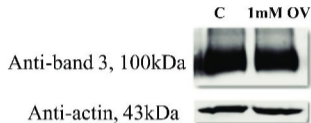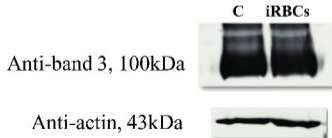

Supplement: S1 Raw images — The lower half of each blot was stained with an anti-actin antibody (to establish that all lanes are loaded equally), while the upper half of each blot was stained with an antibody to the erythrocyte protein, band 3, or phosphotyrosine 8 on band 3, or any (nonspecific) phosphotyrosine. (PDF) [file pone.0242372.s004.pdf]
